# Measurement and hydrodynamic modelling quantifies the mechanical cost of cyprid locomotion

**DOI:** 10.1098/rsif.2026.0344

**Published:** 2026-09-09

**Authors:** Elijah Forstadt, Coco DeFrancesco, Audrey B. Kellogg, Jesse Belden, Beatriz Orihuela, Daniel Rittschof, James C. Bird

**Affiliations:** ^1^ Department of Mechanical Engineering, Boston University, Boston, MA, USA; ^2^ Naval Undersea Warfare Center Newport Division, Newport, RI, USA; ^3^ Duke University Nicholas School of the Environment, Marine Science and Conservation Division, Beaufort, NC, USA

**Keywords:** barnacle cyprid, fluid mechanics, energy expenditure

## Abstract

Barnacle cyprids must locate a settlement site using finite lipid reserves acquired during earlier feeding stages, yet the mechanical cost of their locomotion remains uncertain. Previous estimates of cyprid swimming costs are limited and do not resolve how stroke-scale mechanics, intermittency and temperature shape overall expenditure. Here, we combine high-speed kinematic measurements with an unsteady hydrodynamic model based on the Basset–Boussinesq–Oseen equation to quantify the mechanical cost of swimming in the acorn barnacle *Amphibalanus amphitrite*. Measured stroke-timing distributions inform power estimates from drag, added mass, inertia and the Basset history term. We find that drag dominates mechanical power, with the Basset term providing the next largest contribution. Temperature exerts competing effects: reduced viscosity tends to lower mechanical power, but the observed shortening of power-stroke duration offsets this effect, such that predicted power approximately doubles for every $5^{\circ }C$ increase. Power expenditure during individual strokes ranges from hundreds to more than a thousand picowatts, but locomotion is strongly intermittent, yielding a cycle-averaged mechanical power of only about 30–120 pW, or a few microjoules of mechanical energy per day. Although these estimates are relatively small compared with the cyprid's finite lipid reserves, they could be significant if muscle power conversion is sufficiently inefficient.

## 1. Introduction

Barnacle larvae undergo a final, non-feeding planktonic stage called the cyprid, during which they must locate a suitable surface and settle [1]. This stage is critical because cyprids cannot feed; their survival and successful metamorphosis depend entirely on stored energy reserves accumulated during the earlier nauplius stages [2,3]. Prior work on *Balanus amphitrite* has shown that cyprid energetic condition influences settlement behaviour, metamorphic success and early juvenile performance, underscoring the ecological importance of larval energy reserves [4]. Decades ago, observations by Crisp and others hinted that cyprids have a limited energy budget for swimming and exploration, noting that larvae prevented from settling eventually lose their competence to metamorphose as their reserves dwindle [5,6]. Beyond this point, age and starvation-related degeneration of thoracic musculature leaves cyprids unable to complete metamorphosis even if a suitable surface is encountered [7].

Although it has long been recognized that swimming and exploratory behaviour must draw on these reserves, the mechanical cost of cyprid locomotion remains poorly quantified, particularly in how it varies with environmental conditions such as water temperature. If locomotion consumes a large fraction of their energy, prolonged swimming could lead to exhaustion and larval mortality. Quantifying this mechanical power cost is essential for understanding whether swimming represents a significant fraction of the cyprid's energy budget. Thus, an enduring question in marine larval ecology is how hydrodynamic power demands affect cyprid survival and settlement success. This question becomes especially important as ocean temperatures shift with climate change, since temperature may alter both the mechanical cost of swimming and the timing of settlement-related behaviours.

However, the precise energetic cost of cyprid locomotion is unclear. Most prior studies measured overall metabolic rates or tracked biochemical depletion, such as by loss of lipids over time, without isolating the cost of locomotion from other maintenance needs [3,6]. As a result, a key knowledge gap has been how much of a cyprid's energy budget is expended in locomotion activity and whether this expenditure is substantial enough to limit its lifetime. Advances in high-speed videography and larval biomechanics provide an opportunity to address this gap.

As a first estimate of the mechanical cost of cyprid swimming, consider the power required to translate a sphere of diameter $d_{c}$ through water at speed U. Recent measurements by Lamont & Emlet [1] reported average cyprid swimming speeds ranging from $U=0.64\;{\text{cm s}}^{-1}$ for *Capitulum mitella* (429μm body length) to $U=4.73\;{\text{cm s}}^{-1}$ for *Semibalanus balanoides* (1000μm body length). In the Stokes limit, the required power is $P=3\pi \mu d_{c}U^{2}$, where μ is the dynamic viscosity of water, yielding a mechanical power range of roughly $P=1.7\times 10^{-10}$ to $2.1\times 10^{-8}$ W at a water temperature of $20^{\circ }$C. Although this estimate provides a useful point of reference, it does not capture the multi-modal, unsteady, intermittent and temperature-dependent nature of cyprid locomotion.

**Figure 1.**
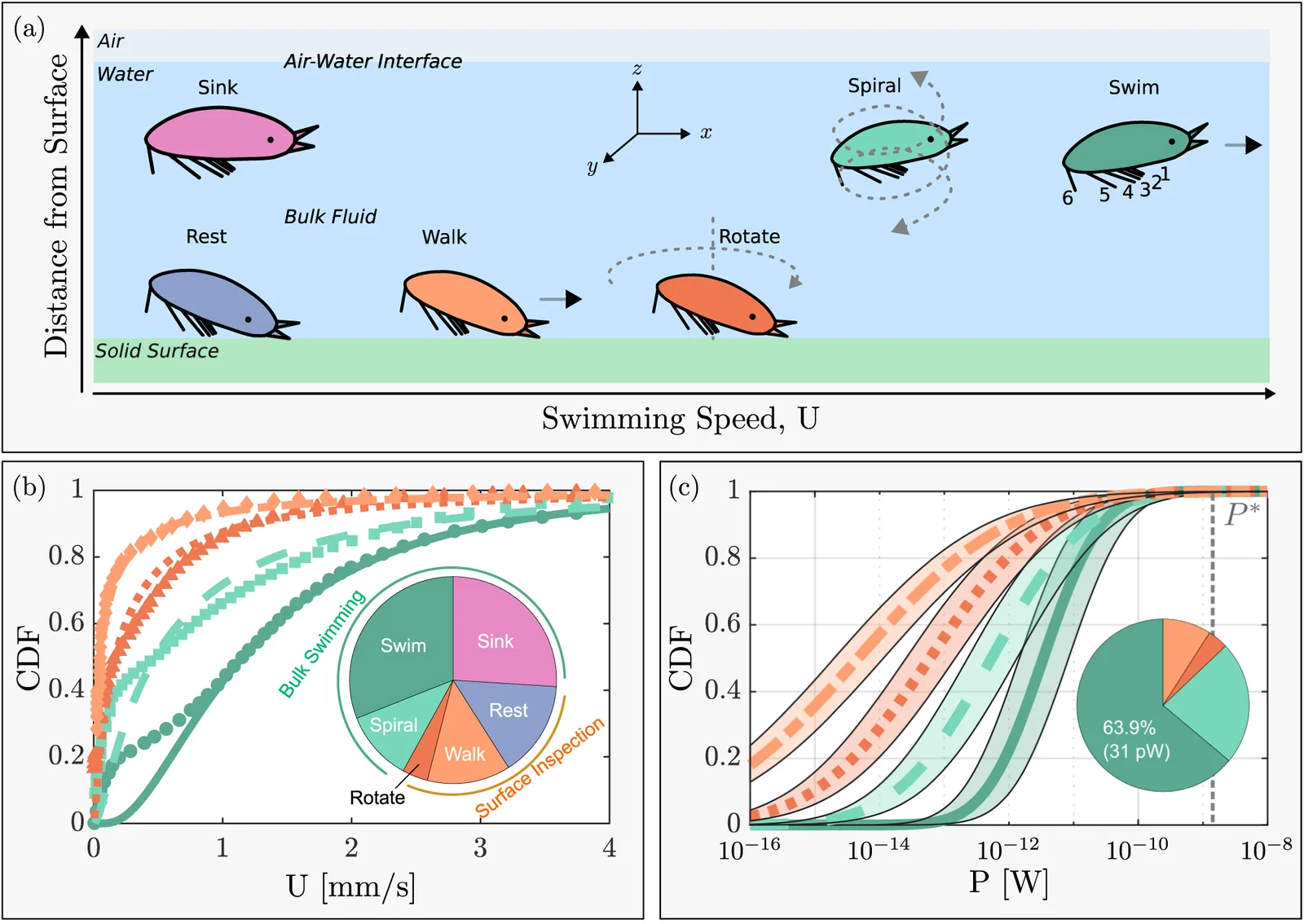
Cyprids exhibit different power expenditures depending on their locomotion mode. (a) Schematic showing the six primary locomotion modes of the cyprid throughout the water column. Cyprids use leg pairs (numbered 1–6) to locomote in the bulk fluid. (b) Digitized cumulative distribution functions (CDFs) of instantaneous speed for the four active locomotion modes of *Amphibalanus amphitrite* cyprids extracted from [8]. Each CDF is fitted with a log-normal distribution. Different line styles denote the different locomotion modes. The fitted parameters $\mu_{U}$ and $\sigma_{U}$ provide the medians ($e^{\mu_{U}}$) and geometric spreads ($e^{\sigma_{U}}$) used in the subsequent power analysis. The inset shows the observed allocation of time among six locomotion modes. (c) CDFs of instantaneous drag power for the four active modes, obtained by mapping the log-normal speed fits of (b) through the relation $P=aU^{b}$. Curves correspond to $20^{\circ }C$ using temperature-dependent water properties, with bounds showing uncertainty from the $C_{D}$ calculation. The inset shows a pie chart of the expected mechanical power contribution from each mode, computed as the time-weighted average power using the mode-specific fractions shown in the inset of (b).

First, cyprids do not move through their environment using a single locomotory behaviour. Rather, they switch among several distinct modes during exploration and settlement, including bulk swimming, spiralling, walking and rotation (figure 1a) [8]. Each mode differs substantially in kinematics, speed and likely energetic cost. During close-range surface exploration, cyprids often walk and rotate using their antennules, advancing in short, deliberate steps [5,9]. By contrast, when moving through the bulk fluid, they swim and spiral with repeated power strokes of their thoracic appendages [1,10] until they encounter shear, after which they respond with rapid swimming to encounter the surface and explore. Additionally, while in the bulk fluid, cyprids alternate between swimming and sinking, resembling the ‘hop-and-sink’ behaviour described for other negatively buoyant zooplankton such as copepods and larval crustaceans, which has been shown to reduce energy expenditure relative to continuous propulsion [11]. Such behaviour may similarly benefit cyprids by minimizing time spent actively stroking, thereby conserving limited energy reserves.

Second, even within a single locomotory mode, cyprids do not move at one constant speed but instead exhibit a broad distribution of velocities (figure 1b). This variability raises the question of what is meant by ‘swimming speed’ in the cyprid literature, as the term is not consistently defined. Some studies report the cumulative distributions of swimming speed and use upper percentiles [8], while others infer from net displacements of set distances [12], or from average velocities over the power stroke [1]. These differences make it difficult to directly compare across studies, and they highlight the importance of explicitly accounting for the inherent variability in cyprid kinematics, rather than relying on a single representative value. This issue is especially important because cyprids typically swim at Reynolds numbers of order unity, Re=ρdcU/μ, where both viscous and inertial effects contribute. In this regime, a purely Stokesian description is insufficient to capture the mechanical cost of locomotion. These considerations motivate our focus on resolving the unsteady stroke dynamics of bulk swimming.

Third, cyprid swimming is inherently unsteady. Cyprids swim by rhythmically beating six pairs of thoracic appendages in an alternating high-drag power stroke and low-drag recovery stroke cycle [13]. During the power stroke, the appendages act like broad paddles; the larva spreads specialized plumose setae that are interconnected by fused setules into a temporary fan with high surface area to push against the water. During the recovery stroke, these setae collapse into a streamlined bundle with minimal drag. This mechanism yields effective drag-based thrust while reducing resistance on the backstroke [1]. Power strokes are characterized by rapid accelerations and decelarations, which give rise to added mass and history forces [14,15], increasing the power cost compared to the Stokes prediction. Thus, unsteady fluid dynamics and drag-based propulsion are at play when cyprid appendages beat in an alternating pattern [16,17]. Recent work on low and intermediate Reynolds active swimmers, including model organisms and simplified propulsors, has clarified how unsteady force generation and added mass effects shape locomotion [18–20]. Such frameworks are highly relevant to cyprids, whose intermittent strokes operate in a regime at the boundary where viscous and inertial effects are important.

Finally, water temperature is expected to influence cyprid swimming in counteracting ways. As temperature increases, the viscosity of water decreases, reducing the drag and potentially lowering the power needed to locomote at a particular speed [21,22]. At the same time, cyprids exhibit faster stroke frequencies at increasing temperatures [23], leading to greater overall mechanical energy expenditure. Although the temperature dependence of muscle contraction in barnacle cyprids has not been directly measured, this faster stroke is likely attributed to general muscle physiology principles; in other ectotherms, muscle contraction is temperature-dependent with a $Q_{10}$ close to 2 [24]. The net effect of temperature on cyprid locomotion cost is therefore not obvious from simple scaling alone, and must account for both changing fluid properties and temperature-dependent stroke kinematics.

Nevertheless, most prior energetic estimates have relied on characteristic or time-averaged swimming speeds combined with Stokes-based drag scaling, and therefore do not capture the multi-modal, unsteady, intermittent and temperature-dependent nature of cyprid locomotion. Here, we address this gap by coupling high-speed kinematic measurements with an unsteady hydrodynamic model to estimate the mechanical energy cost of swimming in barnacle cyprids. Our experiments exploit the distinctive behaviour of cyprids swimming at the air–water interface, where their hydrophobic carapace pins them to the surface and enables quantitative measurement of the characteristic timescales of the stroke cycle. We then use these measurements to quantify the mechanical power cost of locomotion and assess whether this hydrodynamic expenditure is substantial relative to a cyprid's finite energy reserves. Because metamorphosis itself is thought to require of the order of half of a cyprid's stored energy [3], resolving the mechanical cost of swimming is essential for understanding how cyprids budget their limited reserves. More broadly, quantifying the mechanical constraints on cyprid locomotion can improve our understanding of larval ecology and settlement, while also informing strategies for biofouling control.

## 2. Methods and results

### 2.1. Calculating power from steady-state locomotion

To enable us to estimate the mechanical power output expended, we approximate the cyprid as a sphere moving with constant velocity at constant temperature. Typical values for the density and viscosity of water at $20^{\circ }$C are $\rho_{f}=1000\;{\text{kg m}}^{-3}$ and $\mu_{f}=10^{-3}\;\text{Pa s}$, respectively. Previous literature has suggested modelling *A. amphitrite* cyprids as ellipsoids with semi-axes radii $r_{x}\approx 300\mu m$, $r_{y}\approx 125\mu m$, and $r_{z}\approx 60\mu m$, and density $\rho_{c}=1080\;\mathrm{kg}\;m^{-3}$[8]. However, instead of approximating the cyprid as a sphere with equivalent diameter $d_{c}=2(r_{x}r_{y}r_{z}{)}^{1/3}$, we bound the effective frontal area using two spherical diameters, where $d_{c}=2r_{x}$ is the largest span, and $d_{c}=2r_{z}$ is the smallest span. These bounds capture first-order orientation effects on drag and power, providing us with upper and lower estimates on power distributions across swimming modes without fully resolving the cyprid's shape.

Because cyprids in our system swim at intermediate Reynolds numbers, we account for finite Reynolds number effects by modifying the classical Stokes drag formulation using the Schiller–Naumann correlation. We find it convenient in our analysis to express the drag coefficient as a power law using the functional form CD=ARem. Therefore, starting from the Schiller–Naumann correlation [25], we approximate this expression locally by a power law near Re=1, which corresponds to the typical Reynolds numbers of cyprids swimming. The constants A and m are determined by matching both the value and slope of this correlation at Re=1, yielding A=28 and m=−0.9:

(2.1) CD=24Re(1+0.15Re0.69)≈28(ρfdcUμf)−0.9.

Using this coefficient of drag, the power exerted by the cyprid can be written as

(2.2) $$P=\frac{\pi }{8}\rho_{f}d_{c}^{2}U^{3}C_{D}\approx 3.5\pi \rho_{f}d_{c}^{2}{\left(\frac{\mu_{f}}{\rho_{f}d_{c}}\right)}^{0.9}U^{2.1},$$

which captures the slight correction to the Stokes power at Re≈1. Here, note that equation (2.2) has the functional form of

(2.3) $$P=aU^{b},$$

where, by using derived parameters for water at $20^{\circ }$C, $a=2.5\times 10^{-12}\;{[W\;(\mathrm{mm}\;s}^{-1}{)}^{-2.1}]$ and b=2.1. Neglecting inertial effects, the power would be $P=3\pi \mu d_{c}U^{2}$ so that $a=2.9\times 10^{-12}\;[W\;(\text{mm}\;s^{-1}{)}^{-2}]$ and b=2.

Tracking studies by Maleschlijski *et al.* [8] found that instantaneous cyprid speeds are highly variable, ranging from very slow drifts to rapid jumps, with the distribution of burst speeds being strongly right-skewed, as shown in figure 1b. We find that this variability can be approximated by a log-normal distribution:

(2.4) $$F_{U}(U)=\frac{1}{2}\left[1+\mathrm{erf}\left(\frac{\ln U-\mu_{U}}{\sigma_{U}\sqrt{2}}\right)\right]=\frac{1}{2}\left[1+\mathrm{erf}\left(\ln\left({\left(\frac{U}{e^{\mu_{U}}}\right)}^{1/\sigma_{U}\sqrt{2}}\right)\right)\right].$$

We denote the log-normal fit parameters as $\mu_{U}$ and $\sigma_{U}$, so that $e^{\mu_{U}}$ is the median swimming speed and $e^{\sigma_{U}}$ characterizes the multiplicative spread of the distribution. The fitted cumulative distribution function (CDF) is given in equation (2.4), with the fitting parameters tabulated in table 1. To quantify goodness of fit, we computed the Kolmogorov–Smirnov (KS) statistic between the empirical CDFs and the corresponding log-normal fits [26]. Across all locomotion modes, the KS statistics ranged from 0.087 (walking) to 0.216 (spiralling). Figure 1b illustrates the agreement between the log-normal CDF and the empirical cumulative distribution of cyprid swimming speeds. These log-normal distribution fits capture the predominance of moderate speed hops, while still accommodating a long tail of occasional fast bursts up to an observed maximum speed. In a similar manner, other studies have shown that log-normal statistics capture swimming speeds of microorganisms well, in the range of $d_{c}∼10\text{--}200\;\mu m$ [27].

**Table 1.** Log-normal fit parameters for cyprid swimming speed (U) and corresponding steady-state mechanical power expenditure (P) for each locomotion mode of *A. amphitrite*. For each parameter, $e^{\mu }$ is the median and σ is the log-normal shape parameter. E[U] is the mean speed that the cyprid locomotes at. The Kolmogorov–Smirnov (KS) statistic quantifies goodness-of-fit between the empirical data and the fitted CDF. Power was obtained by propagating the fitted speed distributions through the steady-state power model (equation (2.2)). Upper and lower bounds correspond to first-order orientation effects.

mode	time [%]	$e^{\mu_{U}}$ [mm s${}^{-1}$]	$\sigma_{U}$	KS statistic	E[U] [mm s${}^{-1}$]	$e^{\mu_{P}}$ [W]	$\sigma_{P}$	$E[P|P<P^{*}]$ [W]
swim	31	1.15	0.78	0.19	1.56	$1.4\times 10^{-12}$–$8.4\times 10^{-12}$	1.62	$5.4\times 10^{-12}$–$3.1\times 10^{-11}$
spiral	11	0.48	1.28	0.22	1.09	$2.3\times 10^{-13}$–$1.3\times 10^{-12}$	2.68	$5.5\times 10^{-12}$–$3.2\times 10^{-11}$
rotate	4	0.17	1.56	0.18	0.56	$2.6\times 10^{-14}$–$1.5\times 10^{-13}$	3.26	$2.5\times 10^{-12}$–$1.4\times 10^{-11}$
walk	13	0.05	2.01	0.09	0.37	$2.0\times 10^{-15}$–$1.1\times 10^{-14}$	4.20	$1.8\times 10^{-12}$–$1.1\times 10^{-11}$
rest	15	—	—	—	—	—	—	
sink	26	—	—	—	—	—	—	

An advantage of approximating U with a log-normal distribution is that raising a log-normal distributed random variable to a constant power yields another log-normal distribution with scaled parameters. Thus, the CDF for power can be written as

(2.5) $$F(P)=\frac{1}{2}\left[1+\mathrm{erf}\left(\text{ln}\left({\left(\frac{P}{a{\left(e^{\mu_{U}}\right)}^{b}}\right)}^{1/b\sigma_{U}\sqrt{2}}\right)\right)\right].$$

The fitted velocity distribution assigns a small probability to swimming speeds that exceed the maximum values observed in our experiments. To avoid power estimates being influenced by this unphysical tail, we impose an upper bound of $U^{*}\approx 25\;{\text{mm s}}^{-1}$ based on our compiled velocity data. We then calculate the average power expenditure for swimming events below this velocity threshold, as the full distribution would be skewed by extreme events. Using the relation $P=aU^{b}$, $U^{*}$ corresponds to a power of approximately $P^{*}\approx 2.1\;\mathrm{nW}$. This average power below the threshold $U^{*}$ is formally expressed as the conditional expected value $E[P|P<P^{*}]$, which can be evaluated using

(2.6) $$E[P|P<P^{*}]=\exp\left(\mu_{P}+\frac{1}{2}\sigma_{P}^{2}\right)\frac{\Phi (z-\sigma_{P})}{\Phi (z)},$$

where $\mu_{P}=\ln a+b\mu_{U}$, $\sigma_{P}=b\sigma_{U}$ and $z=(\ln P^{*}-\mu_{P})/\sigma_{P}$. Φ(z) is the normal CDF, given by $\Phi (z)=(1/2\pi )\int_{-\infty }^{z}\exp(-t^{2}/2)\;dt$. Assuming constant velocity locomotion, swimming dominates the power expenditure (figure 1c), with an estimated mean of 31pW, motivating us to look more closely at the unsteady motion inherent in the swimming dynamics.

### 2.2. Calculating power from unsteady locomotion

While the steady-state analysis provides a baseline, recent literature has shown that cyprids and copepods undergo frequent accelerations and decelerations during metachronal swimming [1,28], necessitating an unsteady model.

To quantify the mechanical hydrodynamic power exerted by the cyprid in the bulk, we use the Basset–Boussinesq–Oseen (BBO) equation, which describes the hydrodynamic forces acting on a small, rigid and spherical particle accelerating through a viscous fluid [14]. The corresponding power contributions are then obtained by multiplying each force term by the instantaneous swimming speed U. The BBO equation is classically derived under the assumptions of incompressible, Newtonian flow at low Reynolds numbers (Re≪1), with the particle size small compared to the characteristic flow length scales. This framework takes into account the Stokes drag, added mass effects, inertial effects and the Basset force. The Basset force arises because vorticity and momentum diffuse through the surrounding fluid over a finite time, so the viscous boundary layer does not adjust instantaneously to changes in the particle's motion. As a result, the Basset force acts as a history-dependent resistive force that depends on the particle's past acceleration.

However, cyprids of *A. amphitrite* swim at intermediate Reynolds numbers (Re∼1–10), a regime where the low Reynolds number flow assumptions of the classical BBO model are no longer strictly satisfied. To extend the BBO framework to this regime, we replace the classical Stokes drag term with a finite Reynolds number drag correction using the Schiller–Naumann formulation [25], an approach commonly used at moderate particle Reynolds numbers [29–31].

Specifically, the total mechanical hydrodynamic power exerted by the cyprid is modelled as

(2.7) $$P_{\text{total}}=P_{\text{drag}}+P_{\text{added mass}}+P_{\text{inertia}}+P_{\text{Basset}},$$

where

(2.8)Pdrag=π8dc2CD(U)ρfU3,(2.9)Padded mass=π12ρfdc3U˙U,(2.10)Pinertia=π6ρcdc3U˙U(2.11)andPBasset=32dc2πρfμf∫0tU˙(τ)t−τdτ⋅U.

Here, $d_{c}$ is the length of the cyprid prosome and $C_{D}$ is the drag coefficient, as given in equation (2.1). To connect these expressions with measurable stroke kinematics, we introduce a characteristic displacement scale s and power-stroke duration $t_{p}$. We define a characteristic stroke velocity $U_{p}=s/t_{p}$ and acceleration $\dot{U}\approx s/t_{p}^{2}$, and note that $\int_{0}^{t}\frac{d\tau }{\sqrt{t-\tau }}∼2\sqrt{t}$. Approximating the Basset history term at the stroke scale then gives the following characteristic power estimates:

(2.12)Pdrag≈3.5πdc2ρfs3tp3(μftpρfdcs)0.9≈11ρf0.1dc1.1s2.1μf0.9tp2.1,(2.13)Padded mass≈0.26ρfdc3s2tp3,(2.14)Pinertia≈0.52ρcdc3s2tp3(2.15)andPBasset≈3dc2πρfμfs2tp5/2.

These expressions are intended as characteristic stroke-scale power estimates rather than instantaneous power values, obtained by replacing the detailed kinematics with displacement scale s and timescale $t_{p}$. In this way, the scalings retain the key physics of unsteady particle motion at intermediate Reynolds numbers, without requiring a detailed kinematic profile. However, the appropriate choices of the displacement scale s and timescale $t_{p}$, as well as their dependence on temperature, are not yet known. We therefore carry out experiments to quantify these scales and their temperature dependence.

### 2.3. High-speed analysis of cyprid strokes

Batches of *A. amphitrite* cyprid cultures were reared at Duke University Marine Laboratory following established culturing protocols. Upon reaching the cyprid stage, larvae were collected and shipped in sealed test tubes chilled in an ice box. Upon arrival, the samples were stored in a refrigerator at $\text{approximately }3^{\circ }$C. All cyprids used in this study were within eight days of reaching the cyprid stage to ensure a consistent developmental stage and activity level [8].

**Figure 2.**
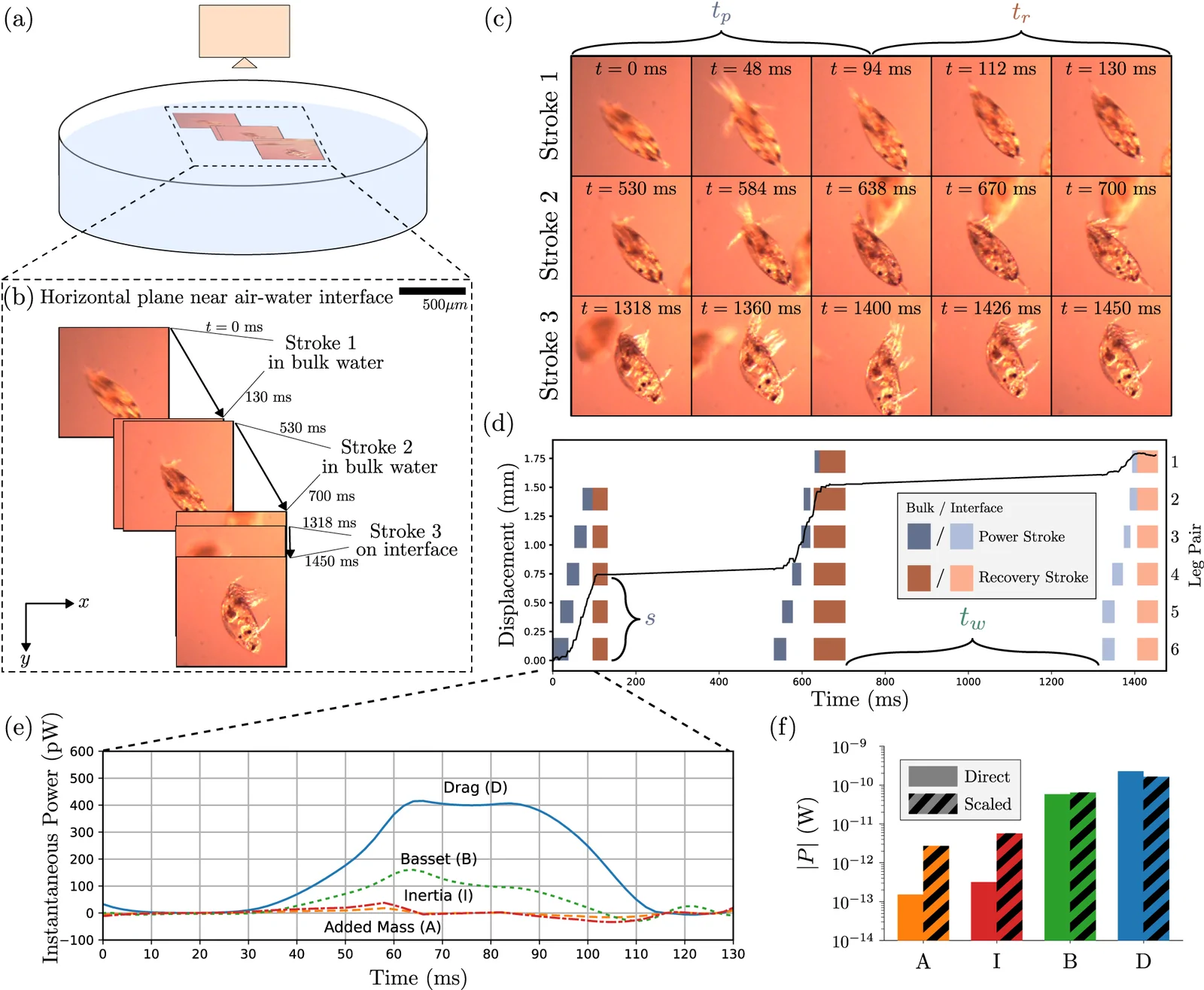
Energetic analysis of successive swimming strokes of *A. amphitrite*. (a) High-speed imaging was used to record *A. amphitrite* cyprids within a test tube. (b) Start and end photographs at the beginning and end of three successive strokes; the cyprid swims two strokes in the bulk, after which it adheres to the air–water interface and takes an additional stroke. (c) Lagrangian view of each of the cyprid's strokes as it goes through the power stroke ($t_{p}$) and the recovery stroke ($t_{r}$). (d) Displacement versus time for three successive cyprid stroke cycles overlaid with stacked bar charts indicating when each leg pair takes a power and recovery stroke. (e) Instantaneous power contributions for Stroke 1 computed using the Basset–Boussinesq–Oseen (BBO) formulation (equations (2.8)–(2.11)). (f) Comparison of stroke-averaged power with scaling estimates (equations (2.12)–(2.15)), demonstrating agreement for drag and Basset contributions.

Videos were captured using a Photron SA-X2 high-speed camera at 500–1000 frames per second, oriented perpendicular to the air–water interface of the specimen (figure 2a). A ruler with 0.5 mm tick spacing was imaged with the focal plane at the interface to estimate the imaging depth. Based on the observed level of focus, cyprids remained within approximately 0.5 mm of the focal plane and typically maintained a similar level of focus during a trajectory, indicating limited out-of-plane motion relative to their body length, such that trajectories were treated as effectively planar. We recorded cyprids swimming in the bulk water near the interface as well as while attached to the air–water interface. Because cyprids are negatively buoyant [8], freely swimming cyprids would sink out of focus during long pauses between strokes. By contrast, surface adhesion allowed individual cyprids to remain in the field of view during extended waiting periods, enabling multiple stroke cycles to be recorded per individual.

For kinematic analysis, a sequence was included once a cyprid within the field of view exhibited a clearly identifiable power stroke. From that point onward, all observable power stroke, recovery stroke and waiting intervals were annotated for as long as the cyprid remained visible or until the video ended. Each included sequence contained at least two strokes, ensuring at least one measurable waiting interval. When the beginning or end of a visible sequence was only partially captured, partial intervals were included only when their durations could be determined unambiguously from the video frames.

To capture low-temperature behaviour, individuals were removed directly from the refrigerator and filmed immediately. The test tube was then allowed to warm naturally to ambient laboratory temperatures ($\text{approx. }21^{\circ }$C) and was subsequently placed in a hot-water bath to reach higher target temperatures. After each temperature adjustment, the bath was gently mixed to ensure a uniform water temperature, followed by a two-minute waiting period to allow turbulence to dissipate prior to recording. Each individual cyprid was recorded at a single ambient water temperature, while multiple stroke cycles were obtained from that individual at that temperature. Thus, temperature-associated differences in stroke timing were evaluated across individuals rather than from repeated measurements of the same individual across temperatures. Accordingly, the temperature trends reported here reflect between-individual comparisons, and a mixed-effects analysis assessing the contribution of inter-individual variability is provided in appendix A.

A representative sequence of three successive stroke cycles is shown in figure 2b. The first two strokes occur below the air–water interface, while the third occurs at the interface itself. This motion reveals three fundamental timescales that characterize cyprid swimming. The power stroke, $t_{p}$, is identified as a rapid burst of forward propulsion initiated by full extension of the thoracic appendages. The recovery stroke, $t_{r}$, follows as the appendages retract in preparation for the next cycle (figure 2c). Net horizontal displacement was measured across the three-stroke sequence (figure 2d), showing that most of the displacement was achieved over the power stroke, with minimal drift during the recovery. After the recovery stroke, the cyprid remains stationary for a waiting time, $t_{w}$, before initiating the next stroke. Here, the waiting time, $t_{w}$, is defined as the interval between the end of a measurable recovery stroke and the onset of the next measurable power stroke within the same visible sequence.

We use the displacement curve from the first stroke cycle to compute the instantaneous hydrodynamic power contributions using the full unsteady model (equations (2.8)–(2.11)), decomposed into drag, Basset, added-mass and inertial contributions (figure 2e). Although surface adhesion was useful experimentally for keeping individuals in the field of view during extended waiting periods, the hydrodynamic model is intended to represent swimming in the bulk fluid. Surface recordings are therefore used primarily to resolve stroke timing, while the power calculations are taken to represent bulk swimming. We then compare these direct results with the characteristic power estimates in equations (2.12)–(2.15) (figure 2f). These estimates capture the dominant contributions to mechanical power, but overpredict the added-mass and inertial components.

**Figure 3.**
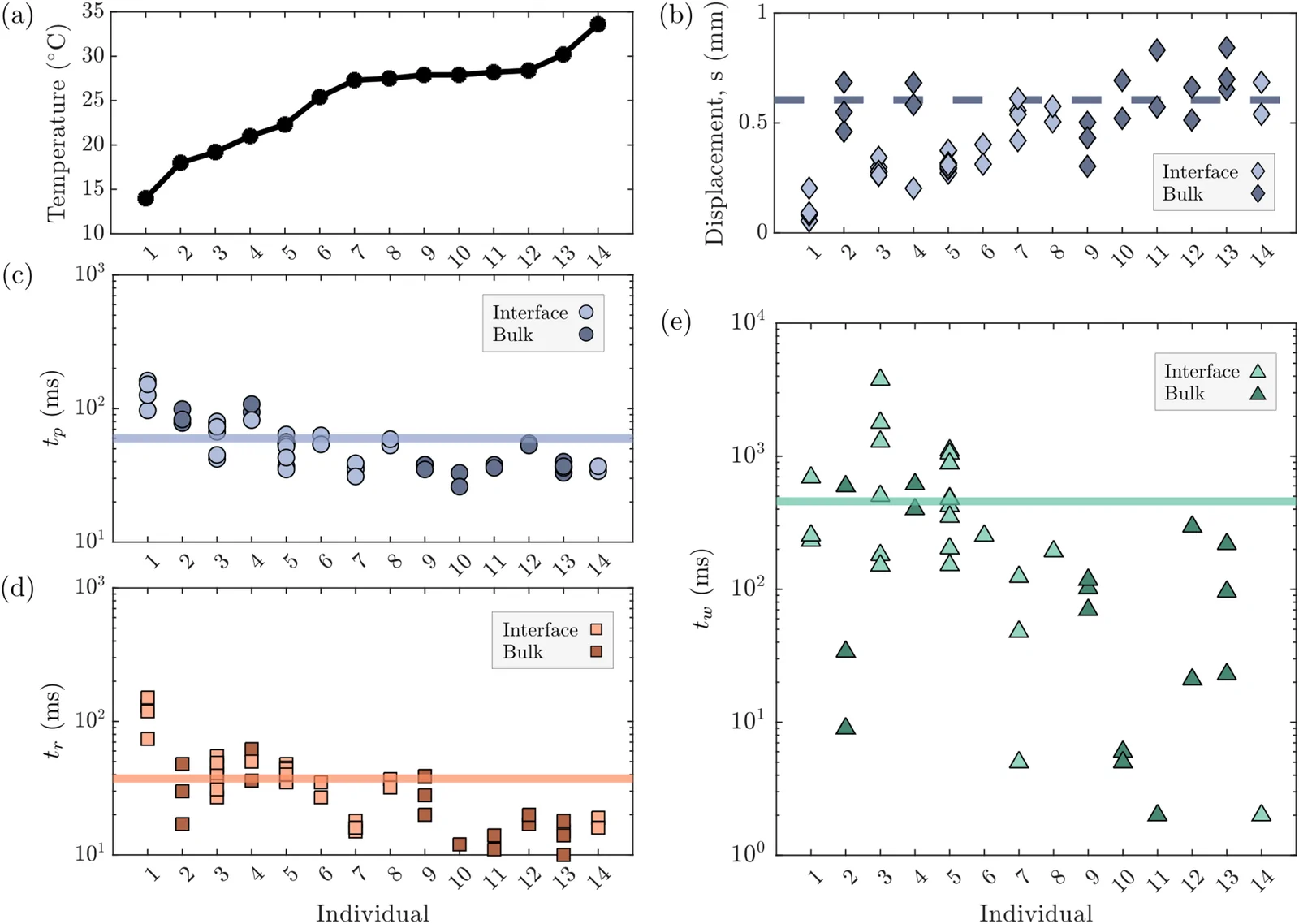
Experimental stroke timing and displacements of 14 individual *A. amphitrite* cyprids, each taking multiple strokes. Each individual was measured at a single water temperature, and the set of individuals spans a broad temperature range; individuals are ordered by increasing temperature, and this ordering is identical across all panels. (a) Water temperature was measured for each individual. (b) Displacement, s, in millimetres over the power stroke measured from high-speed videos. Each point represents a single stroke, colour-coded by swimming depth (light: interface; dark: bulk). The dashed line refers to the mean displacement among all bulk strokes. (c–e) Power stroke duration ($t_{p}$), recovery-stroke duration ($t_{r}$) and waiting time ($t_{w}$) for the same individuals, respectively. Each point is similarly coloured by swimming depth. The solid line denotes the mean among all measurements; dashed lines indicate means below and above $20^{\circ }C.$.

To quantify variation in stroke kinematics across individuals and temperatures, we manually annotated over 40 individual strokes across 14 cyprids, drawn predominantly from a single batch (figure 3). Across the experimental temperature range, power-stroke displacement remained relatively consistent, particularly for strokes occurring in the bulk fluid, indicating that the distance advanced per stroke varied less strongly than the stroke timing (figure 3b). By contrast, the temporal components of the stroke cycle showed distinct patterns (figure 3c–e). Most notably, the power-stroke duration, $t_{p}$, decreased with increasing temperature, consistent with the mixed-effects analysis reported in appendix A. Recovery-stroke durations, $t_{r}$, also appeared shorter at higher temperatures, although this trend was not analysed separately here. The waiting time, $t_{w}$, by contrast, exhibited substantially greater scatter across individuals and strokes. These measurements provide the experimental basis for the temperature-dependent timing relations used in the subsequent power analysis, while table 2 summarizes the pooled timing distributions using two-parameter Gamma fits.

**Table 2.** Fitted Gamma distribution parameters for power, recovery and waiting durations of *A. amphitrite* cyprids, pooled across 14 to $34^{\circ }$C. The Gamma distribution is characterized by shape parameter α and scale parameter θ, with mean αθ and variance $\alpha \theta^{2}$. Values in parentheses indicate 95% confidence intervals. The KS statistic quantifies goodness-of-fit between empirical and fitted distributions.

variable	shape (α)	scale (θ)	mean [ms]	$\sqrt{\mathrm{Var}}$ [ms]	KS statistic
$t_{p}$	5.01 (3.41–7.35)	11.9 (7.97–17.9)	${\bar{t}}_{p}=59.8$	26.7	0.14
$t_{r}$	2.45 (1.69–3.55)	15.3 (10.1–23.1)	${\bar{t}}_{r}=37.4$	23.9	0.12
$t_{w}$	0.56 (0.39–0.81)	768 (442–1334)	${\bar{t}}_{w}=431$	575	0.07

## 3. Discussion

### 3.1. Faster strokes at higher temperatures can outweigh reduced viscous resistance

**Figure 4.**
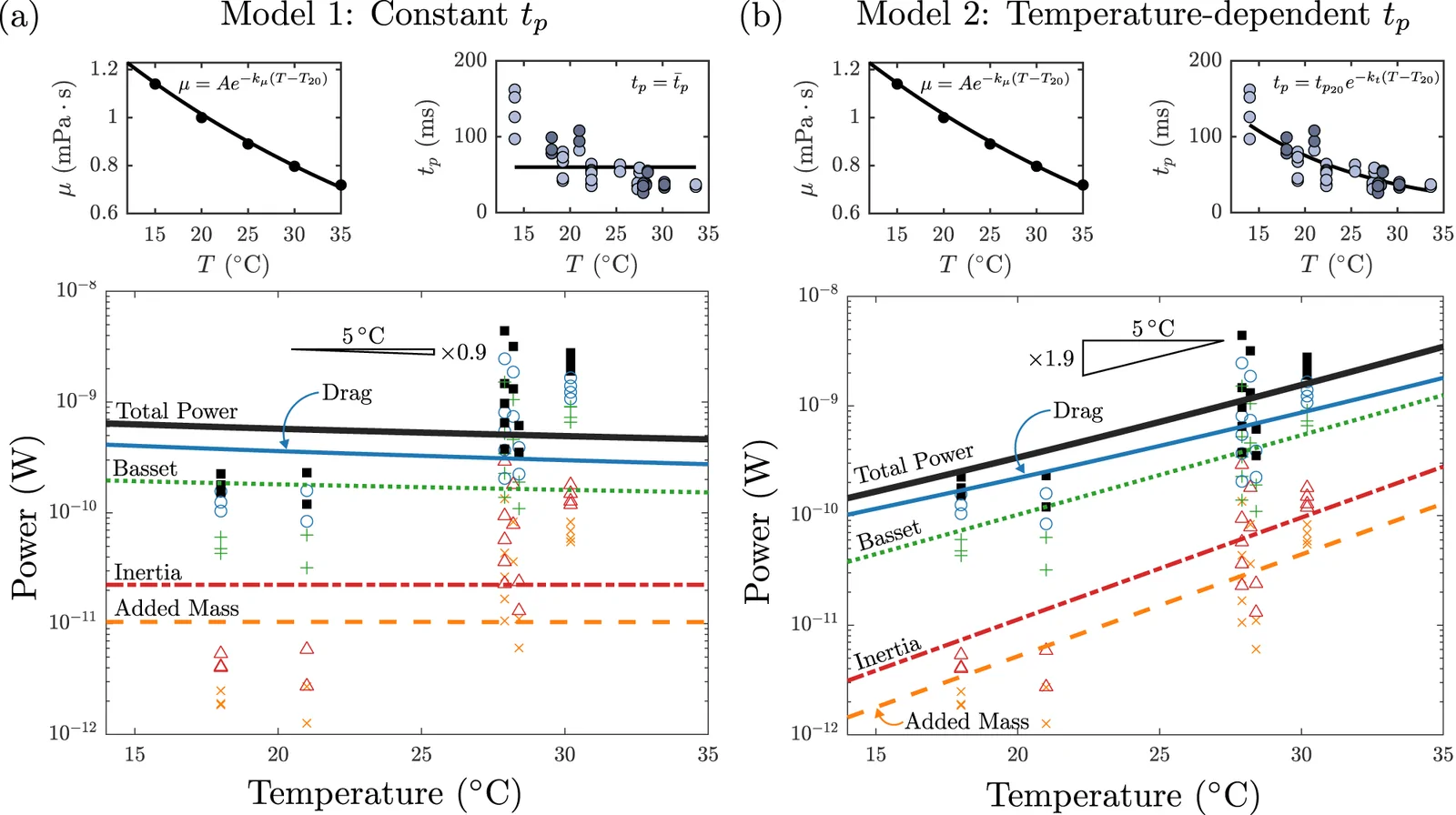
Temperature dependence of swimming power predicted from hydrodynamic modelling. Power expenditure of cyprid swimming was derived from the BBO framework with temperature-dependent fluid properties. Small panels show model inputs, while larger panels compare predicted total power with experimental measurements. (a) Viscosity-only model: water viscosity varies with temperature according to an exponential fit to tabulated values, while the mean power-stroke duration $t_{p}$ is held constant. (b) Coupled physiological-fluid model: both viscosity and stroke duration vary with temperature, with $t_{p}$ described by an exponential fit to experimental measurements. Lines denote model predictions and symbols indicate experimentally inferred power values for strokes in the bulk (equations (2.12)–(2.15)).

To examine how temperature influences the mechanical cost of a single swimming stroke, we applied the characteristic BBO power model (equations (2.12)–(2.15)) across the observed experimental temperature range. Figure 4 compares the resulting model predictions with experimentally inferred stroke powers for subsurface swimming strokes. In addition to the total predicted power, we also evaluated the individual drag, Basset, added-mass and inertial contributions. The experimentally inferred total stroke power tends to increase with temperature, suggesting that the temperature dependence cannot be explained by fluid property changes alone.

To interpret this trend, we express each power contribution in the reduced form

(3.1) $$P_{i}∼\mu_{f}^{\alpha }t_{p}^{\beta },$$

where α and β depend on the specific hydrodynamic contribution. This form highlights the two dominant temperature-dependent inputs in the model: the fluid viscosity $\mu_{f}$ and the power-stroke duration $t_{p}$. Over the range considered here, the density of water varies only weakly compared with its viscosity, so the leading-order temperature dependence is captured by $\mu_{f}$ and $t_{p}$. Near atmospheric pressure, water density changes only modestly over this range, whereas viscosity decreases substantially with increasing temperature [32,33].

We first consider a viscosity-only model in which stroke kinematics are held fixed and only fluid properties vary with temperature. Over the temperature range of our experiments, the viscosity of water is well approximated by an exponential relation:

(3.2) $$\mu_{f}∼\mu_{20}e^{-k_{\mu }(T-20)},$$

where $\mu_{20}=1.02\;\text{mPa s}$ and $k_{\mu }=0.024{\;}^{\circ }C^{-1}$, consistent with tabulated data (figure 4 inset).

Substituting this into the power scaling yields

(3.3) $$\ln P_{i}∼-\alpha k_{\mu }(T-20)+\mathrm{const}.,$$

and predicts that mechanical power should decrease monotonically with increasing temperature. Using dataset-averaged kinematics ($t_{p}=59.8\;\mathrm{ms}$, s=0.605mm), the total predicted stroke power decreases from approximately 630pW at $15^{\circ }C$ to 490pW at $30^{\circ }C$ (figure 4a; table 3). This decrease reflects the reduced hydrodynamic resistance associated with the drop in water viscosity from about 1.14mPa s at $15^{\circ }C$ to about 0.80mPa s at $30^{\circ }C$.

**Table 3.** Predicted power-stroke swimming speed and mechanical power expenditure for *A. amphitrite* cyprids at representative cold (15${}^{\circ }$C) and warm (30${}^{\circ }$C) temperatures. Results are shown for a constant $t_{p}$ model and a temperature-dependent $t_{p}(T)$ model. Here, ${\bar{U}}_{p}$ is the mean swimming speed during the power stroke and ${\bar{P}}_{p}$ is the corresponding mean mechanical power expenditure during the power stroke.

		constant $t_{p}$ model		temperature-dependent $t_{p}$ model	
	μ [mPa s]	${\bar{U}}_{p}$ [mm s^–1^]	${\bar{P}}_{p}$ [pW]	${\bar{U}}_{p}$ [mm s^–1^]	${\bar{P}}_{p}$ [pW]
$15^{\circ }$C	1.14	10.1	630	5.6	170
$30^{\circ }$C	0.80	10.1	490	16.4	1550

However, this viscosity-only prediction does not match the experimental trend. Instead, the measured stroke powers increase with temperature, indicating that changes in stroke timing must also be considered. In our data, $t_{p}$ is well described by an exponential decrease with temperature:

(3.4) $$t_{p}(T)∼t_{p20}e^{-k_{t}(T-20)},$$

with $t_{p20}=75.2\;\mathrm{ms}$ and $k_{t}=0.071{\;}^{\circ }C^{-1}$. Substituting both $\mu_{f}(T)$ and $t_{p}(T)$ into the scaling yields

(3.5) $$\ln P_{i}∼-(\alpha k_{\mu }+\beta k_{t})(T-20)+\mathrm{const}.$$

When the experimentally observed temperature dependence of $t_{p}$ is incorporated into the model, the predicted stroke power increases with temperature despite the lower viscosity (figure 4b). In this model, the effect of faster strokes outweighs the reduction in viscous resistance. Equivalently, for each $5^{\circ }C$ increase, the viscosity-only model predicts a modest reduction in stroke power (approx. 0.9×), while the coupled model predicts an approximate doubling (approx. 1.9 times).

Whether power ultimately increases or decreases with temperature depends on the relative magnitudes of these two contributions. In particular, power is expected to increase with temperature when

(3.6) $$\frac{k_{t}}{k_{\mu }}\geq -\frac{\alpha }{\beta }.$$

In other words, the shortening of the stroke timescale with temperature must be strong enough to offset the viscosity-driven decrease in hydrodynamic resistance.

Table 3 summarizes the predicted power expenditures for *A. amphitrite* at representative low and high temperatures. Under the constant $t_{p}$ model, warming reduces the predicted stroke power. Under the temperature-dependent $t_{p}$ model, warming instead increases both characteristic stroke speed and power substantially, consistent with the trend observed in the experiments.

### 3.2. Swimming intermittency strongly affects energy budget

The single-stroke analysis above quantifies the mechanical power expended during an active power stroke, but cyprids do not swim continuously. Instead, swimming consists of repeated cycles containing a power stroke, a recovery stroke and a waiting interval. To account for this intermittency, we model the distributions of $t_{p}$, $t_{r}$ and $t_{w}$ using pooled Gamma fits across all temperatures (table 2). These fits provide a compact description of the observed timing variability, with KS statistics below 0.14 for all three variables. Assuming that cyprids expend energy primarily during the power stroke, the energy expended over that period is

(3.7) $$E_{\text{stroke}}={\bar{P}}_{p}\;t_{p},$$

where ${\bar{P}}_{p}$ is the mean power during the power stroke. Thus, the average power, accounting for swimming intermittency, is the stroke energy divided by the total cycle duration:

(3.8) $$\bar{P}=\frac{{\bar{P}}_{p}\;{\bar{t}}_{p}}{{\bar{t}}_{p}+{\bar{t}}_{r}+{\bar{t}}_{w}},$$

where ${\bar{t}}_{p}$, ${\bar{t}}_{r}$ and ${\bar{t}}_{w}$ are the mean durations of the power stroke, recovery stroke and waiting interval, respectively.

This expression shows that recovery and waiting intervals reduce the cycle-averaged power relative to the instantaneous power of the active stroke. The waiting interval is particularly important because its mean duration is substantially longer than either $t_{p}$ or $t_{r}$ (table 2). Thus, although a single stroke may require appreciable mechanical power, the average energetic cost over extended locomotor sequences is strongly moderated by intermittency.

To examine how this intermittency interacts with temperature, we combine the pooled estimates of $t_{r}$ and $t_{w}$ with either a constant or temperature-dependent description of $t_{p}$. This allows us to compare representative cold and warm conditions while isolating the effect of the observed shortening of the power-stroke duration with increasing temperature. The predicted averaged swimming speeds and mechanical power expenditures are summarized in table 4. Under the constant timing model, $\bar{P}$ decreases from 71pW at $15^{\circ }C$ to 56pW at $30^{\circ }C$. When the measured temperature dependence of $t_{p}$ is incorporated, however, the trend reverses, and the predicted average power increases from 31pW to 113pW. Intermittency, therefore, substantially reduces the overall energetic cost of locomotion, but when the observed temperature dependence of $t_{p}$ is included, it does not eliminate the predicted increase in power expenditure at higher temperature.

**Table 4.** Predicted averaged swimming speed and mechanical power expenditure for *A. amphitrite* cyprids at representative cold (15${}^{\circ }$C) and warm (30${}^{\circ }$C) temperatures. Results are shown for a model with constant $t_{p}$, $t_{r}$ and $t_{w}$, and for a model with temperature-dependent $t_{p}(T)$ and pooled $t_{r}$ and $t_{w}$. Here, $\bar{U}$ denotes the cycle-averaged swimming speed over the full stroke cycle.

		constant $t_{p},t_{r},t_{w}$ model		$t_{p}(T)$, constant $t_{r},t_{w}$ model	
	μ [mPa s]	$\bar{U}$ [mm s^–1^]	$\bar{P}$ [pW]	$\bar{U}$ [mm s^–1^]	$\bar{P}$ [pW]
$15^{\circ }$C	1.14	1.15	71	1.05	31
$30^{\circ }$C	0.80	1.15	56	1.20	113

This framework also helps reconcile the present unsteady estimates with the simpler steady drag-based estimates. Steady estimates inferred from literature-scale swimming speeds (figure 1) are of the order of 10–30pW, whereas the present unsteady model predicts the much larger mechanical powers over the power stroke. Once recovery and waiting times are included, however, these values are reduced to averaged powers between about 31 and 113pW, much closer to the steady range. The two approaches, therefore, describe different aspects of cyprid locomotion: steady drag-based models based on mean swimming speed approximate the longer-time average mechanical cost of sustained locomotion, but they do not capture the elevated power demands associated with rapid acceleration during individual power strokes. By contrast, the unsteady framework resolves these peak stroke-scale power demands while still recovering average power values once intermittency is accounted for. In this sense, steady estimates can approximate average swimming cost, but underestimate the mechanical demands of active propulsion.

### 3.3. Interfacial swimming may reduce locomotion cost

Although the energetic analysis above was interpreted primarily in terms of swimming in the bulk fluid, cyprids can swim up to the air–water interface, be drawn to their sides and be suspended by surface tension [5,34]. This raises the question of whether interfacial swimming might reduce the energetic cost compared to bulk swimming. Two possible mechanisms are considered here: reduced cost of regaining height lost during passive sinking and reduced hydrodynamic resistance owing to partial immersion.

One possible energetic advantage is the reduced cost of regaining height lost during passive sedimentation. In the bulk, cyprids are slightly denser than seawater and frequently exhibit the alternating swimming and sinking modes described above. Because the cyprid density exceeds that of the surrounding fluid by $\Delta \rho =\rho_{c}-\rho_{f}$, the net downward force is of order

(3.9) $$F_{g}∼\Delta \rho gV.$$

The characteristic mechanical power associated with repeatedly recovering from sinking therefore scales as the net downward force times a representative vertical speed:

(3.10) $${\overset{―}{P}}_{\mathrm{support}}∼F_{g}\;{\overset{―}{u}}_{\mathrm{sink}}∼\Delta \rho gV\;{\overset{―}{u}}_{\mathrm{sink}},$$

where ${\overset{―}{u}}_{\mathrm{sink}}$ is the mean sinking speed. Using representative values for *A. amphitrite* cyprids reported by Maleschlijski *et al.* [8], with $V\approx 9.4\times 10^{-12}\;m^{3}$ based on the ellipsoidal body dimensions and ${\overset{―}{u}}_{\mathrm{sink}}\approx 1.2\times 10^{-3}\;m\;s^{-1}$ based on the measured sinking speed yields ${\overset{―}{P}}_{\mathrm{support}}∼10^{-12}$–$10^{-11}\;W$. Although this is smaller than the characteristic locomotion powers estimated above, it is sufficiently large that suppressing repeated sinking could modestly reduce the overall energy budget.

A second possible advantage is that only part of the body is submerged while the cyprid is adhered to the air–water interface. As a crude geometric approximation, the hydrodynamic force and power scales may therefore decrease in proportion to an effective submerged fraction ϕ, giving

(3.11) $$P_{\mathrm{interface}}∼\phi P_{\mathrm{bulk}},$$

with ϕ∼1/2 for an approximately half-immersed body. Under this approximation, the drag-dominated power identified above would likewise be reduced by a comparable factor. This estimate should be interpreted cautiously, however, since free surface hydrodynamics are not determined solely by submerged area: drag on partially immersed bodies also depends on immersion depth, wetting, contact-line geometry and interfacial deformation [35]. At the same time, the cyprid velocities measured here are far below the minimum capillary–gravity wave speed for water ($c_{\min}\approx 0.23\;m\;s^{-1}$), so classical steady wave-making drag is not expected [36,37]. Any interfacial modification to energetic cost is therefore more likely to arise from capillary support, wetting effects and changes in wetted area than from wave generation.

Taken together, these considerations suggest that locomotion at the air–water interface could reduce energetic cost, most clearly by reducing the need to repeatedly regain height lost during passive sinking. Partial immersion may also reduce hydrodynamic resistance, although quantifying that effect more precisely would require a dedicated model for cyprid motion at the air–water interface.

### 3.4. Long waiting times within swimming can allow passive descent

Can passive descent arise within ordinary swimming intermittency? To answer this, we compare the measured waiting times with the characteristic timescale for sinking by one body length. Using a characteristic body length $d_{c}∼0.6\;\mathrm{mm}$ and sinking speed ${\bar{u}}_{\mathrm{sink}}$, we estimate $t_{\mathrm{sink}}∼d_{c}/{\bar{u}}_{\mathrm{sink}}\approx 0.5\;s$. Thus, pauses of order 0.5s are sufficient for appreciable passive descent, consistent with the empirical 0.6s threshold previously used to distinguish swimming from sinking [8].

The waiting times measured here refer specifically to inter-stroke intervals within swimming trajectories, rather than arbitrary periods of inactivity. These intervals are reasonably described by a Gamma distribution (table 2), with fitted parameters α=0.56 and θ=768ms. For $t_{0}=0.5\;s$, the probability that a waiting interval exceeds this threshold is $P(T>t_{0})\approx 28.7\%$. These longer pauses account for a disproportionate fraction of the total waiting time:

$$\frac{\int_{t_{0}}^{\infty }tf(t)\;dt}{\int_{0}^{\infty }tf(t)\;dt}\approx 74.8\%.$$

Thus, intervals long enough for appreciable passive descent are not rare tail events, but a substantial component of the observed intermittency. This suggests that passive descent need not be treated as wholly separate from intermittent swimming, but can arise naturally from the same broad waiting-time distribution.

### 3.5. Swimming costs consume only a small fraction of available energy reserves

*A. amphitrite* cyprids possess finite internal energy reserves, with reported lipid-derived stores of roughly 3–6mJ over the cyprid lifetime [3]. To place our results in an ecological context, we compare these reserves with the mechanical energy expenditure predicted by the locomotion model.

Using the measured stroke timing statistics, the constant $t_{p},t_{r},t_{w}$ model predicts daily mechanical expenditures of $6.2\;\mu {\text{J d}}^{-1}$ at $15^{\circ }C$ and $4.8\;\mu {\text{J d}}^{-1}$ at $30^{\circ }C$, while the temperature-dependent $t_{p}$ model predicts $2.7\;\mu {\text{J d}}^{-1}$ and 9.8μJ d^–1^, respectively. These values are obtained by extrapolating the measured stroke-cycle statistics over 24 h, and thus represent the mechanical cost of continuously repeating the observed locomotor cycle. Relative to a 3–6mJ reserve, this corresponds to approximately 0.05–0.33% of the available energy per day.

This comparison should be interpreted cautiously, since our model estimates mechanical work and power, whereas the reported reserve values reflect stored chemical energy. We therefore do not assume that metabolic expenditure equals mechanical work. Because muscle converts only a fraction of chemical energy into external mechanical work, the true metabolic cost of swimming is expected to exceed the mechanical work estimated here [38]. Consequently, our calculations should be interpreted as providing a lower bound on the energetic cost of swimming, and the mechanical work reported here should not be interpreted as the total metabolic cost. The energetic significance of swimming may therefore be greater than suggested by the mechanical work estimates alone.

Even with this caveat, the predicted mechanical cost of routine swimming remains modest relative to the total stored reserves. This suggests that cyprids can sustain repeated bouts of locomotion and exploration while retaining substantial reserves for maintenance, settlement and metamorphosis. At the same time, the higher expenditure predicted at elevated temperature in the temperature-dependent $t_{p}$ model suggests that warming could accelerate reserve depletion, even if swimming remains mechanically feasible in absolute terms.

## 4. Conclusion

Using high-speed video measurements and an unsteady BBO framework with a finite-Reynolds-number drag correction, we quantified the characteristic mechanical power associated with *A. amphitrite* cyprid swimming. We find that drag is the dominant contribution to the power budget, while the Basset history term exceeds the added-mass and inertial contributions.

The temperature dependence of this mechanical cost reflects two competing effects: increasing temperature lowers fluid viscosity, but also shortens the power-stroke duration and increases stroke speed. The net result is that temperature-dependent changes in stroke kinematics outweigh the viscosity-driven reduction in hydrodynamic resistance, such that the coupled model predicts an approximate doubling of power expenditure for every $5^{\circ }C$ increase. By incorporating measured power stroke, recovery and waiting times, we further showed that cyprid locomotion is strongly intermittent, yielding an averaged mechanical power of about 30–120pW, or a few microjoules of mechanical energy per day. The broad distribution of waiting times further suggests that sinking can arise naturally within intermittent swimming, rather than as a wholly separate behavioural mode.

Compared with reported cyprid energy reserves, these results suggest that the direct mechanical cost of routine swimming is modest relative to the total stored energy available during the cyprid stage, although this comparison should be interpreted cautiously as the present model predicts mechanical rather than metabolic expenditure. Swimming in the water column is therefore unlikely, by itself, to be the dominant energetic limitation on cyprid survival. More broadly, our analysis suggests that locomotion at the air–water interface may further reduce energetic cost by suppressing passive sedimentation and lowering the effective submerged body area. However, the total energetic burden of the cyprid stage also includes behaviours not quantified here, including exploratory walking and surface inspection during settlement [39], as well as settlement-related interactions such as deactivation of host defences in symbiotic species [40]. Metamorphosis may also contribute substantially to reserve depletion, but that cost was not quantified here.

## Supplementary Material



## Data Availability

All data supporting the findings and figures in this paper are available as supplementary material. Supplementary material is available online [41].

## Appendix A: Mixed-effects analysis of temperature dependence in stroke timing

To evaluate whether the observed temperature dependence could be explained solely by differences among individuals, we fitted a linear mixed-effects model of the form $t_{p}∼\mathrm{Temp}+(1\;|\;\mathrm{Individual})$, treating individual identity as a random intercept. The fixed temperature effect was strongly negative (slope $\beta =-4.76\;\mathrm{ms}{\;}^{\circ }C^{-1}$, 95% CI: −6.28 to −3.24, $p=8.98\times 10^{-8}$), indicating a systematic decrease in power-stroke duration with increasing temperature. Across the observed temperature range, this corresponds to an estimated change of approximately 93 ms in mean $t_{p}$.

By comparison, the between-individual standard deviation of the random intercepts was 12.9 ms, while the within-individual standard deviation was 12.3 ms (ICC≈0.52). Although the inter-individual variability is substantial, the magnitude of the temperature-associated shift is considerably larger than differences among individuals. These results indicate that the observed trend is unlikely to be accounted for by baseline individual-to-individual variability alone, although the experimental design does not allow a definitive separation of temperature effects from possible temperature-structured sampling differences among individuals. Because each individual was measured at a single temperature, we interpret this result as evidence of a population-level association with temperature rather than a strictly within-individual causal response.
